# Multiple components of statistical word learning are resource dependent: Evidence from a dual-task learning paradigm

**DOI:** 10.3758/s13421-021-01141-w

**Published:** 2021-03-17

**Authors:** Tanja C Roembke, Bob McMurray

**Affiliations:** 1 Jaegerstrasse 17-19, Institute of Psychology, RWTH Aachen University, 62062 Aachen, Germany; 2 Departments of Psychological and Brain Sciences, Communication Sciences and Disorders, and Linguistics, University of Iowa, Iowa City, IA USA

**Keywords:** Cross-situational word learning, Dual systems accounts of learning, Propose-but-verify, Dual-task paradigm, Implicit learning

## Abstract

It is increasingly understood that people may learn new word/object mappings in part via a form of statistical learning in which they track co-occurrences between words and objects across situations (cross-situational learning). Multiple learning processes contribute to this, thought to reflect the simultaneous influence of real-time hypothesis testing and graduate learning. It is unclear how these processes interact, and if any require explicit cognitive resources. To manipulate the availability of working memory resources for explicit processing, participants completed a dual-task paradigm in which a cross-situational word-learning task was interleaved with a short-term memory task. We then used trial-by-trial analyses to estimate how different learning processes that play out simultaneously are impacted by resource availability. Critically, we found that the effect of hypothesis testing and gradual learning effects showed a small reduction under limited resources, and that the effect of memory load was not fully mediated by these processes. This suggests that neither is purely explicit, and there may be additional resource-dependent processes at play. Consistent with a hybrid account, these findings suggest that these two aspects of learning may reflect different aspects of a single system gated by attention, rather than competing learning systems.

**Supplementary Information:**

The online version contains supplementary material available at 10.3758/s13421-021-01141-w.

## Introduction

Many domains of learning and memory have been dominated by debates about the specific mechanisms of learning that underlie a given learning problem. These debates have contrasted different representational assumptions, such as whether a set of categories is learned by acquiring rules, prototypes, or exemplars (e.g., Richler & Palmeri, 2014; J. D. Smith, 2014); they have contrasted architectural aspects of learning such as whether something is declarative or procedural (Squire, 1992); and they have asked whether a given problem requires one system or two (e.g., Ashby et al., 1998; Edmunds et al., 2018). These debates have often played out in the context of fairly straightforward supervised learning tasks in which on each trial either the correct response is available or the subject receives feedback.

In contrast, work on language learning has traditionally focused on less constrained, unsupervised forms of learning, in which listeners are simply exposed to a series of inputs and left to their own devices to identify structure. Such learning has been motivated by the natural ecology of language acquisition in which children are thought to receive little explicit instruction or direct feedback to support learning. Here too, there have been extensive debates between clashing mechanisms (e.g., Pinker & Ullman, 2002; Rumelhart et al., 1986). However, in the type of laboratory tasks often deployed in this domain (and presumably in real-world language learning), learners are completely unconstrained and often do not even have a task. Consequently, a number of researchers have begun to consider the possibility that learners engage multiple learning mechanisms simultaneously (e.g., Endress & Bonatti, 2016; Feldman et al., 2013; Roembke & McMurray, 2016).

For example, children may acquire auditory categories (speech sounds) of their language by tracking the distributional statistics of specific cue values (e.g., formant frequencies, voice onset) and identifying clusters; however, they may simultaneously be identifying larger chunks (e.g., words) and using them to separate categories (e.g., the fact that the /ih/ sound occurs in *milk* and *spin* while the /eh/ sound occurs in distinct words like *bread* and *friend*) (Feldman et al., 2013), or they may use the visual referents of words (e.g., *pet* and *pit*) in a similar fashion (Räsänen & Rasilo, 2015). This kind of hybrid is certainly not unique to language – the SUSTAIN model of categorization (Love et al., 2004), for example, includes both unsupervised and supervised learning. Nevertheless, the emphasis on unsupervised tasks in language highlights its possibility.

In these hybrid learning models, it is critical to start to understand the cognitive processes that underlie specific components of learning: Does each component require attention, feedback, cognitive resources, and so forth? The present paper offers a first step in this direction. Specifically, we focus on a popular statistical learning paradigm for learning word-object-associations without feedback – cross-situational learning. As we describe, initial debates have started assuming a single mechanism approach, and research has contrasted statistical from more propositional accounts of learning. This debate is ongoing, but it has left us with clear behavioral indices of several processes that play out simultaneously during learning – processes that may loosely correspond to an implicit/explicit distinction. Here, we use a dual-task procedure to ask if either of these processes require cognitive resources. In this, we were loosely inspired by dual-task work on visual categorization (Waldron & Ashby, 2001), though we are not arguing for an explicit multiple-learning systems architecture (which may not even be supported in visual categorization, e.g., Lewandowsky et al., 2012).

### Cross-situational word learning

In the last decade, an explosion of work has asked how learners use statistical learning to acquire the mapping between words and their referents (e.g., Dautriche & Chemla, 2014; Escudero, Mulak, & Vlach, 2016; Roembke & McMurray, 2016; Scott & Fisher, 2012; Smith & Yu, 2008; Yurovsky, Yu, & Smith, 2007). This has been motivated by the fact that children master a huge quantity of words relatively quickly but with little overt teaching or feedback. Consequently, researchers have sought unsupervised mechanisms that do not require a teaching signal. Work in this area has converged on a specific statistical learning paradigm known as cross-situational word learning (Yu & Smith, 2007).

Cross-situational word learning was originally intended as a laboratory model that captures two critical assumptions about how children learn the meanings of new words: (1) they often do so without explicit feedback, and (2) in any naming situation, there are many possible referents for a novel word. Cross-situational learning proposes that even if any single naming event was ambiguous, learners can acquire new words by combining information across encounters to identify the correct word-object-mappings (Siskind, 1996; Yu & Smith, 2007).

In a standard cross-situational learning experiment, participants see several objects and hear one or more novel words. Each word is presented an equal number of times and each picture appears equally often. Across trials, the target object and word are consistently paired, whereas foil objects are randomly selected across trials. Crucially, this means that object competitors never co-occur with a word as often as its referent. In this procedure, the referent of a word may be unclear on any one trial, particularly early, when no words are known. However, the learner can determine the correct mappings by using the co-occurrence statistics *across trials*. Importantly, participants are never told that it is their task to use the co-occurrence of words and objects to extract the correct mappings, and they get no feedback as to their performance.

Ample empirical evidence demonstrates that both adults and children can learn novel words in this paradigm (Dautriche & Chemla, 2014; Fitneva & Christiansen, 2015; Koehne et al., 2014; Roembke et al., 2018; Roembke & McMurray, 2016; L. B. Smith & Yu, 2008; Suanda et al., 2014; Trueswell et al., 2013; Yu & Smith, 2007). Thus, this is a viable mechanism early in development when children observe their surroundings in the absence of consistent feedback, but also for word learning during later years (Fitneva & Christiansen, 2015; Roembke et al., 2018; Suanda et al., 2014).

### Contributions of multiple learning mechanisms in cross-situational word learning

Originally, cross-situational word learning was framed as an associative process of statistical learning (Yu & Smith, 2007). Each time a word is heard and an object is seen, their connection gets strengthened (or their co-occurrence statistic gets a bump). Since the correct object is consistently paired with its word across situations or trials, this association grows, whereas associations between the word and other objects remain weak (since these do not co-occur as frequently). This can occur even in the absence of feedback. Later at test, people use the underlying association matrix to respond in the moment and select the correct object for the word that was heard (McMurray et al., 2012; Yu & Smith, 2012). Importantly, this learning process is supposed to be implicit, and awareness of the word-object-mappings is not necessary to acquire them (Wang, 2020; Yu & Smith, 2007).

In contrast, Trueswell et al. (2013; see also Medina, Snedeker, Trueswell, & Gleitman, 2011) have argued that participants could employ a more explicit strategy that operates within a single trial, which they term “propose-but-verify.” When presented with an ambiguous trial (a word for which they do not know the referent), learners form a hypothesis as to the referent of the word. On subsequent trials, this “proposed” word-object-mapping is then verified (if it is consistent with the objects on that trial), or discarded if contradictory evidence is encountered (e.g., if that object is not present; Koehne et al., 2014; Medina, Snedeker, Trueswell, & Gleitman, 2011; Trueswell et al., 2013). Critically, rather than maintaining multiple partial hypotheses (or associations) for a given word, in a propose-but-verify system, learners maintain only a single hypothesis.

In support of this claim, Trueswell et al. (2013) present evidence that the accuracy for an individual word is largely predicted by the accuracy on the prior encounter with that word: when the learner was incorrect on the last encounter (trial) with a word, they were largely at chance on the current encounter. According to propose-but-verify, this is because their last-encounter hypothesis was clearly incorrect, and they had to abandon it and start over (Trueswell et al., 2013). Additionally, consistent with the proposal that cross-situational learning uses explicit processes, Trueswell et al. (2016) reported evidence that participants could distinguish between less and more informative learning situations, indicating awareness of their learning.

The way that these two opposing accounts – associative learning and propose-but-verify – have been described roughly maps onto the functional properties of implicit and explicit forms of learning. An associative or statistical learning account is more consistent with implicit learning that gradually accumulates statistics over trials. In contrast, propose-but-verify (as described by Trueswell et al., 2013) is explicit and rational, mapping more closely onto rule-based, symbolic or inferential forms of explicit learning.

Subsequent studies challenged a pure propose-but-verify account. Dautriche and Chemla (2014) conducted similar analyses but took into account whether the foil object was also present on a current trial. When this was done, there was evidence for gains in performance even after an incorrect trial. Perhaps most notably, Yurovsky, Fricker, Yu, and Smith (2014) showed that after a first phase of learning, words that had not been learned (they were still at chance) were still learned faster in a second phase. This suggests that even if learners had the wrong hypotheses for a word, they had still acquired partial statistical evidence to help with future learning. Finally, Roembke and McMurray (2016) presented eye-tracking evidence showing that even as learners may be clicking on the correct object, they still maintain evidence for alternative meanings. These studies do not rule out the sort of processes posited by propose-but-verify. Indeed, in many of these studies, there is still a very strong influence of whether subjects responded accurately on the last encounter. However, they suggest that in addition to this kind of processing, there may be an associative learning process as well.

These findings raise the possibility of a hybrid model (Roembke & McMurray, 2016; Roembke et al., 2018; see also McMurray et al., 2012; Yurovsky & Frank, 2015). That is, these hypothesized learning mechanisms represent the function of two distinct processes that operate in parallel during word learning and likely interact. Under this kind of hybrid, the gradual implicit accumulation of statistics across trials occurs in parallel to more real-time, possibly explicit, processes (Roembke & McMurray, 2016; Roembke et al., 2018; see also McMurray et al., 2012; Yurovsky & Frank, 2015). Importantly, in order to apply in the context of statistical learning, both of these systems must operate without feedback.

### Trial-by-trial analyses: Markers of propose-but-verify and gradual learning

A critical source of evidence for this hybrid is trial-by-trial analyses of the learning curve that have identified indices that reflect propose-but-verify or gradual (associative) learning processes (Roembke et al., 2018; Roembke & McMurray, 2016). For example, we conducted a standard cross-situational learning paradigm and used trial-by-trial analyses to examine performance. As in Trueswell et al. (2013), we examined the effect of the accuracy the last time the target was encountered (which we term the *last-encounter-accuracy* effect) as a marker of something like an inferential (propose-but-verify) process. However, we also simultaneously estimated the statistical effect of how much exposure the subject had with that word (up to that point in the experiment), which we term the *target-exposure* effect. This allowed us to estimate the effect of raw number of exposures to a word *over and above* the learners’ evolving inferential knowledge (represented by the last-encounter-accuracy effect). That is, we can estimate each simultaneously, controlling for any possible collinearity.

We found significant main effects of both target-exposure and last-encounter-accuracy, consistent with a hybrid account. Moreover, the effect of last-encounter-accuracy interacted with target-exposure, such that the effect of last-encounter-accuracy increased at later points during training. This suggests some interaction between systems such that increasing statistical evidence allows learners to make better trial-by-trial inferences later. We later extended these findings to children and to mappings between non-linguistic sounds and referents, suggesting these indices are robust in the learning curve (Roembke et al., 2018).

### Implicit and explicit mechanisms

This statistical approach offers the ability to isolate the simultaneous effects of gradual learning and or inferential processes like propose-but-verify. What is not clear is the degree to which either of these processes are explicit (requiring awareness and cognitive resources) or implicit. Indeed, the open-ended nature of the cross-situational word-learning task leaves this question quite open. On the one hand, in a standard cross-situational word-learning experiment with adults, participants are typically told that they are supposed to acquire the word-object-mappings they are exposed to. In addition, response times during testing are unlimited. These characteristics may encourage the use of explicit processes. On the other hand, participants are not specifically instructed to use co-occurrence statistics of words and objects. Similarly, the majority of cross-situational word-learning experiments have used unfamiliar objects as referents, which makes it more difficult to engage reasoning processes as they do not have other names (e.g., Roembke et al., 2018; Roembke & McMurray, 2016; Smith & Yu, 2008; Yurovsky et al., 2007). These factors could result in the use of implicit processes, and are indeed why a statistical account was proposed as the mechanism of learning.

Thus, it is not straightforward to identify the contributions of explicit and implicit learning mechanisms in cross-situational learning. A critical hallmark of explicit processing is the use of cognitive resources. This has been well developed within work on categorization (e.g., Waldron & Ashby, 2001). The idea is that if explicit memory resources are engaged with a second task, learners are more likely to rely on implicit learning processes.

Wang (2020) leveraged this paradigm for a more direct investigation of explicit and implicit memory representations in cross-situational word learning. In this study, participants completed a categorization task as a cover; at the same time, they were also exposed to word-object-mappings cross-situationally. The extent to which the explicit or implicit system was used for learning was deduced from the presence/absence of a relationship between participants’ confidence and word-learning performance. Results suggested that participants used the explicit memory system when known objects were used as referents, but that the implicit one was engaged when words had to be mapped onto novel, unfamiliar objects where no verbal encoding had been possible (Wang, 2020). The extent to which the implicit and/or explicit system is used during word learning may thus depend on a number of situational characteristics, such as the amount of referential ambiguity, familiarity of the visual referents, presence of a cover task (task instructions) or time pressure to respond (Wang, 2020; Yurovsky & Frank, 2015).

Nevertheless, this does not address the issue of whether specific sub-components of learning – the gradual associative or inferential processes identified above – are each explicit or implicit. This is important because if these processes do dissociate along these lines, it would suggest the involvement of two distinct learning systems (e.g., Ashby & Maddox, 2005). However, if neither are explicitly mediated or if both are, this would be consistent with a single system mediated by general cognitive operations like working memory or attention. While the original framing of each process seems to support a straightforward implicit/explicit contrast, there are several reasons this remains an open question.

First, the last-encounter-accuracy effects – which were intended to support an explicit propose-but-verify account – could be explained via other means. This possibility is highlighted by the fact that pigeons – a species unlikely to engage in inferential processing – showed an effect of last-encounter-accuracy in a task that was developed to mimic human word learning (Wasserman et al., 2015). An alternative conceptualization of the last-encounter-accuracy effects can derive from the dynamic associative account of McMurray et al. (2012; also see Roembke & McMurray, 2016). This account argues that associations do not just count raw co-occurrence – objects that are selected show larger gains. When a learner selects the target object, its associative link to the word should be increased more than the unselected (but present) objects. This then increases the learners’ probability of being correct again on a subsequent trial (assuming the prior selection was correct). This “boost” from selection need not be large; in-the-moment competition processes can amplify small differences in the underlying associative strength, making behavior look more accurate than the stored mappings. Thus, the last-encounter-accuracy effect may not derive entirely from explicit processes.

Second, a recent study by Warren et al. (2020) investigated cross-situational word learning in patients with hippocampal amnesia. Importantly, the hippocampus has often been thought necessary for the formation of explicit, verbalizable rules as well as for “memory in the moment.” Warren et al. (2020) found that amnesic patients could acquire the word-object-mappings; however, their learning was slower and less stable. This also suggests explicit processes are not strictly necessary for learning. However, the fact that learning was poorer suggests that while cross-situational word learning can be accomplished with purely associative or implicit processes (mediated by neocortical or procedural learning systems), explicit learning (mediated by hippocampal systems) also contributes. However, as Warren et al. (2020) did not conduct trial-by-trial analyses, it is not clear if the specific sub-components of cross-situational word learning contribute to the more gradual associative portion, the inferential portion, or both.

### Current study

To summarize, existing studies of cross-situational word learning are loosely consistent with explicit and implicit approaches to learning. Moreover, the hybrid proposed by Roembke and McMurray (2016), suggests two processes with distinct markers in the learning curve (last-encounter-accuracy and target-exposure). However, a crucial question is whether any *specific* aspect of cross-situational learning is an explicit process. The most likely candidate would be the last-encounter-accuracy effect (and by extension, proposing and verifying), though other processes may contribute to the last-encounter-accuracy effect as well.

Here, we take the same dual-task approach of Wang (2020) to investigate the role of explicit and implicit systems in learning word-object-mappings:^1^ The present experiment limits access to general cognitive resources with a dual-task paradigm to provide insights into how important explicit processes are for cross-situational word learning. Specifically, we assess the degree to which the last-encounter-accuracy or the gradual associative learning effects are explicit, and how effective unsupervised word learning is under such circumstances.

In this study, all participants completed the same cross-situational word-learning task in which accuracy was recorded on each trial. Simultaneously, they participated in either a verbal working memory task that required the maintenance of several numbers (*high-load*) or one that only required remembering one number (*low-load*). Here, we operationally define working memory as the “ensemble of components of the mind that hold a limited amount of information temporarily in a heightened state of availability for use in ongoing information processing” (p. 1163; Cowan, 2017). The working memory task we used did not require the manipulation of the presented content. Working memory was manipulated between subjects, as we were worried that having subjects participate in the word-learning task twice could create confounds (e.g., the words learned in the first task could interfere with word learning in the second task).

Critically, we leveraged the trial-by-trial approach of Roembke and McMurray (2016) to ask which components of learning use explicit resources. To do this, we go beyond thinking of effects like the last-encounter-accuracy effect as merely indicating one learning system or another. Rather, we use it as a more continuous measure of how much a given aspect of learning is operative as a function of memory load. That is, we ask if load numerically reduces or enhances each of the two trial-by-trial effects on the learning curve. This design allowed us to address the following questions:

1. How does cross-situational word learning change when participants have limited working memory resources?
2. Is the effect of last-encounter-accuracy modulated and/or dependent on the availability of working memory resources?
3. Is the effect of target-exposure modulated and/or dependent on the availability of working memory resources?
4. Is the effect of load on performance fully mediated by the last-encounter-accuracy and target-exposure effects?

At the broadest level, it is likely that an increased working memory load may delay word learning, as this would limit the ability of explicit systems to make in-the-moment inferences. Moreover, we predicted that this effect would be more evident at the end of the experiment than at the beginning (c.f., Roembke & McMurray, 2016). At the start, participants’ accuracy might be more driven by chance and association, and less by explicit processing. Yet, as they learn more word-object-mappings (including the auditory word forms and visual representations), they can use working memory resources in the moment to make referent selections. This is based on our prior work, which shows a stronger last-encounter-accuracy effect later in the learning curve.

Considering our trial-by-trial effects, if the last-encounter-accuracy effect is mediated by an explicit learning system, then it should be reduced – but perhaps not disappear – under load. Such a partial reduction would indicate that people do remember what word they last clicked on from trial to trial, but a lack of complete elimination would suggest that other processes could contribute to this benefit. A similar prediction might be made for the gradual learning effect. If explicit processing is necessary for this effect (e.g., statistical learning is gated by attention: Toro et al., 2005), then it should be reduced under high memory load.

Putting these together, we see two possible patterns. First, under some dual-systems accounts of learning, implicit and explicit processes compete, such that one tends to dominate at any given point in learning or for any given task (e.g., Ashby & Maddox, 2005; Maddox et al., 2003; Waldron & Ashby, 2001). Under a competitive model, a reduction in the last-encounter-accuracy effect should be paired with an increase in the target-exposure effect (gradual statistical learning). Alternatively, if both effects derive from different aspects of the same learning system (McMurray et al., 2012), and this learning system uses some explicit resources, we might expect both trial-by-trial indices to be reduced similarly in the presence of load.

Finally, we ask if the effects of load on learning are fully mediated by the two trial-by-trial indices, or if there is a main effect of load even after we account for these effects. Such a main effect would point to explicit processes that are not fully captured by propose-but-verify or gradual associative learning.

### Method

#### Participants

Eighty-seven monolingual, native speakers of English participated. Participants were students at the University of Iowa and received course credit as compensation. Four participants in the low-load condition were excluded, as they did not appear to be complying with the instructions and had runs of trials in which they clicked on the same screen location regardless of the word.^2^ Moreover, one participant in the high-load condition was run on the incorrect randomization list due to experimenter error. Finally, one participant in the low-load condition was excluded from all analyses for below-chance performance on the memory task (see *Results*). In summary, six participants were excluded prior to analysis, leaving 81 participants in the final data set for analysis (n _high load_ = 40; n _low load_ = 41). All exclusions were made before data analysis was started.

#### Stimuli

Participants learned 12 word-referent pairs. Words were two-syllable, phonologically legal CVCV pseudo-words (Table 1). Phonological overlap between words was minimized to facilitate participants’ learning. Auditory stimuli were recorded in a neutral carrier phrase by a female native speaker of English. Five exemplars of each word were used during the experiment to add natural variation in speech. Exemplars were edited to remove elements that were not part of the word (e.g., jaw clicks). Finally, all recordings were normalized and 50 ms of silence was added to their beginning and end. Referents were photographs of highly infrequent objects and thus likely unfamiliar to participants, presented on a white background. Mappings of referents and words were random for each participant.

#### Design

**Fig. 1:**
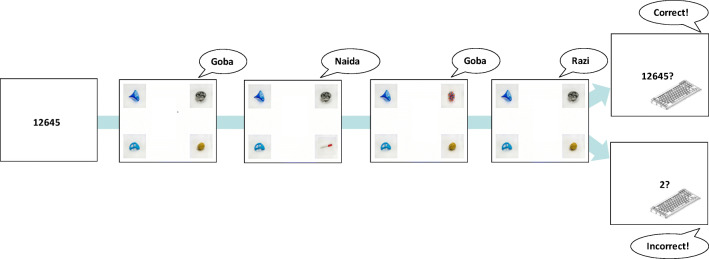
Graphic depiction of the experimental procedure in *high-load* (upper box) and *low-load* (lower box) conditions. Feedback was given in both the *high-load* and *low-load* memory tasks, but never on cross-situational word-learning trials

This study employed a dual-task paradigm in a between-subjects design manipulating difficulty of the secondary task (Fig. 1): Participants completed two tasks interleaved with each other: a cross-situational word-learning task and a working memory task. Participants were randomly assigned to one of two conditions that affected the working memory task (high/low load). The cross-situational learning task was identical between conditions. Random assignment was based on whether the participant number was odd or even.

During cross-situational word learning trials, participants were presented with four objects and a single target word. Trials always included the target object and three randomly chosen foils. Foils were selected from the available pool without replacement to avoid the possibility of one foil object accidentally co-occurring with the target word at high rates. The location of each object was randomized across the four possible locations on each trial. There were 360 cross-situational word learning trials, separated into three blocks of 120 trials. Trials were randomly ordered within a block.

During the memory task, participants saw five numbers. This number was selected to fill up working memory, but not make it impossible to store additional information (Woods et al., 2011). Numbers were randomly selected out of all possible digits (0–9); no number was repeated within a set of five.

The memory task was interleaved among four cross-situational word-learning trials: Participants saw the numbers, then completed four cross-situational learning trials, and then responded to the memory task, before moving on to the next set of trials. Order of trials within each set of four trials was random, thus the same word could be repeated within a set of four trials. In the high-load condition, participants reported the five numbers in the correct order; in the low-load condition, they reported the number of even numbers in the set. Since participants knew in advance what their task would be, in the low-load condition, they could simply count the number of even numbers and retain a single value. Thus, the only difference between the high-load and low-load conditions was the quantity of numbers maintained in memory while completing the word-learning trials. Overall, there were 90 memory trials.

#### Procedure

Participants were instructed that their task was to discover which object goes with what word, and that, on each trial, they should select the object that they believed mapped onto the word they heard. They were also given the instructions for the memory task (which differed by condition). Participants were informed that while they were expected to perform well on the memory task, the word-learning task was of primary interest.

At the start of each cross-situational word learning trial, four pictures were presented in the four corners of a 19-in. monitor operating at 1,280 × 1,024 resolution. Simultaneously, a small blue circle appeared at the center of the screen. Participants were given 1,050 ms to inspect the objects. Subsequently, the circle turned red, cueing the participant to click on it to cue the auditory stimulus. When the participant clicked on it, the circle disappeared, and the word was played via headphones. Participants then clicked on one of the four objects to end the trial. Again, the response selection was self-paced. Participants never received feedback on cross-situational learning trials.

At the beginning of a set of four cross-situational learning trials, participants were reminded of the instructions for their memory task; they were then shown the five numbers for 1 s each. After the completion of four word-learning trials, they then responded to the memory task by typing a number or string of numbers on the keyboard. Participants were given feedback, indicating whether they had made the correct response on the memory task: a “bell” sound indicated a correct response, whereas a “buzz” sound indicated an incorrect response.

### Results

First, we analyzed performance on the memory task to check the validity of the dual-task manipulation. As part of the dual-task design, it was essential to document that participants were engaged in the memory task to ensure that the high-load indeed required more working memory resources. Second, we analyzed participants’ trial-by-trial performance during word learning to determine which components of performance were affected by the memory load. A coarser analysis of mean performance in the word earning task is available in Supplement S1 (Online Supplemental Materials, OSM).

#### Memory task

For the digit span task (high-load condition), accuracy was calculated for each number position separately, and then the average was taken. For instance, if the digits to be remembered were 4, 1, 9, 5, 7 and a participant reported 4, 2, 9, 7, 5, that trial would be considered 40% correct (only the first and third digit were correct). For the low-load condition, participants’ single numeric response was evaluated against the correct one.

We adopted a conservative criterion to guarantee that participants complied with the dual-task design. A participant was considered to be guessing their average accuracy was below 40%. None of the participants in the high-load condition were excluded based on this, while a single low-load participant was excluded (accuracy: 37.5%).

**Fig. 2:**
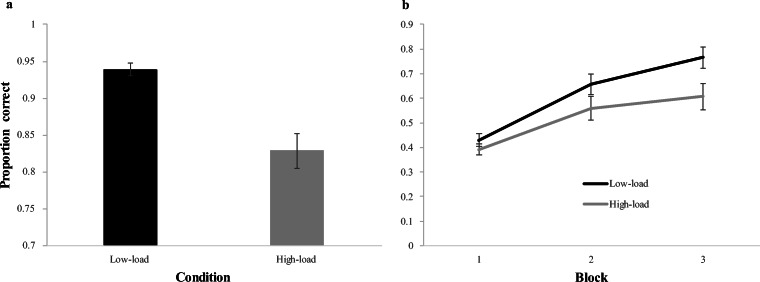
(**A**) Performance on the memory task by condition. Error bars indicate standard error of the mean (SEM). (**B**) Word-learning accuracy across blocks as a function of load condition*.* Each block represents 120 cross-situational learning trials. Error bars indicate SEM

Average accuracy for each condition is presented in Fig. 2A. Participants in the low-load condition averaged above 90% correct, whereas participants in the high-load condition averaged just above 80% correct. This difference in performance is not surprising, as the *high-load* task was designed to be more difficult and had a much lower chance level than the *low-load* task. To confirm this pattern statistically, we ran a one-way ANOVA on condition (between subjects); the DV was empirical logit transformed accuracy^3^ in the memory task. As suspected, participants in the low-load condition were significantly more accurate than in the high-load condition (*F*(1, 79) = 23.97, *p* < 0.001, η_p_^2^ = 0.233).

#### Word learning

Figure 2B shows learning performance as a function of load. Participants in the low-load condition again out-performed participants in the high-load condition; this was particularly evident by the end of training (see Supplement S1 (OSM) for an analysis of accuracy as a function of block and condition without the trial-by-trial factors).

Our core analysis asked whether the availability of working memory resources moderated the effects of last-encounter-accuracy and target-exposure. Importantly, if the effect of last-encounter-accuracy in part reflects participants’ memory of their previous response, this process should be hindered in the high-load condition and we should observe a smaller benefit of having selected the correct object on the last encounter with that word. In contrast, the effect of target-exposure is seen as a marker of more gradual implicit statistical learning during cross-situational word learning. If this is the case, it should be less affected by the availability of working memory resources, as it is presumed to be driven by associative or statistical processes that do not require domain-general cognitive resources.

To investigate this statistically, binomial mixed-effect models were implemented in R (version Ri386 3.6.3; R Core Team, 2014) using the lme4 (Bates & Maechler, 2009) and nlme (Pinheiro et al., 2020) packages. The fixed effects were (1) last-encounter-correct (1/0; whether a participant had selected the correct object the last time s/he was exposed to the word [effect of last-encounter-accuracy]); (2) target-exposure (how often the target word had appeared up to that trial, log-scaled); (3) condition (low-load/ high-load); (4) all of their possible interactions. To provide a measure of effect size for each reported effect, we calculated odds ratios by exponentiating the raw coefficients.

To identify the random effects, a series of nested models were compared to find the model with most complex random effect structure required to fit the data (Matuschek et al., 2017); we used a forward selection approach, evaluating a more complex model against a less complex model using the chi-square test of model comparison (detailed results of this are available in Supplement S2 (OSM)). The most complex model that still converged included a random effect of subject and target object but no random slopes:

1 $$ \mathrm{accuracy}\sim \mathrm{last}-\mathrm{encounter}-\mathrm{correct}\ast \mathrm{target}-\mathrm{exposure}\ast \mathrm{condition}+\left(1|\ \mathrm{subject}\right)+\left(1|\ \mathrm{target}\ \mathrm{object}\right) $$

The main effects of last-encounter-correct and target-exposure were significant (last-encounter-correct: B = 1.30, SE = 0.03, Z = 40.24, *p* < 0.001, odds ratio = 3.67; target-exposure: B = 0.74, SE = 0.02, Z = 32.35, *p* < 0.001, odds ratio = 2.10). This is a standard finding and parallels earlier studies (Roembke et al., 2018; Roembke & McMurray, 2016). These two effects suggest that, first, participants were more likely to be accurate on a current trial if they had been also correct on a previous encounter with that word; and, second, that they were more likely to be accurate when they had encountered the target more often. Critically, the presence of target-exposure in the model controls for position in the learning curve on the effect of last-encounter-correct (e.g., the effect cannot be driven by the fact that early trials tended to have last-encounter-correct = 0, while late trials tended to have last-encounter-correct = 1). The interaction of last-encounter-correct and target-exposure was also significant (B = 0.46, SE = 0.04, Z = 10.49, *p* < 0.001, odds ratio = 1.58); this was because the effect of last-encounter-accuracy was higher at the end of the experiment (c.f., Roembke & McMurray, 2016). The presence of these standard effects suggest that word learning was not qualitatively different from previously conducted experiments that have employed this analytic strategy (e.g., Roembke & McMurray, 2016; Roembke et al., 2018).

**Fig. 3:**
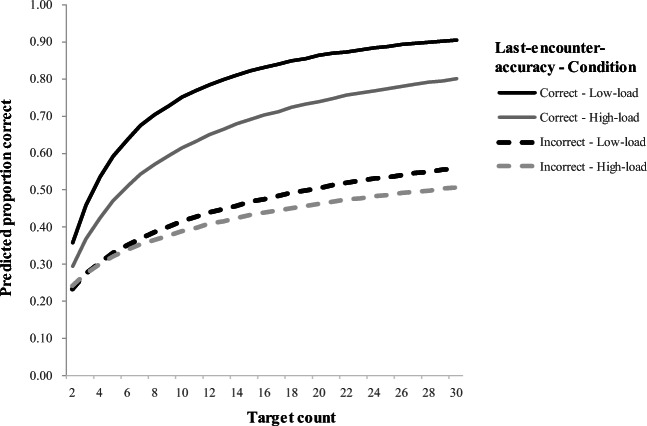
Model predictions for effect of target-exposure after splitting data by last-encounter-accuracy (correct/incorrect) and condition (low-load/high-load; based on predictions generated by model (1)

Importantly, the interaction between last-encounter-correct and condition was significant (B = -0.56, SE = 0.06, Z = -8.65, *p* < 0.001, odds ratio = 0.57). The effect of last-encounter-accuracy differed in the two conditions: Participants in both conditions were equally likely to be incorrect on a current trial if they had been incorrect on their last encounter with the target word. However, participants in the low-load condition had a larger effect of last-encounter-accuracy than participants in the high-load condition (Fig. 3). In addition, the two-way interaction of target-exposure and condition was also significant (B = -0.16, SE = 0.05, Z = -3.56, *p* < 0.001, odds ratio = 0.85). This was due to the fact the slope of target-exposure was steeper under low-load than high-load conditions (Fig. 3). The three-way interaction was not significant (*p* = 0.167).

To investigate these significant two-way interactions further, data were split by condition, and the models were re-run without the effect of condition and its associated interactions (after Bonferroni correction, α = 0.0125). The main effect of last-encounter-accuracy was significant in both the high-load (B = 1.02, SE = 0.03, Z = 22.79, *p* < 0.001, odds ratio = 2.77) and the low-load conditions (B = 1.57, SE = 0.05, Z = 33.75, *p* < 0.001, odds ratio = 4.81). However, the reduced coefficient and effect size in the high-load condition are consistent with a smaller effect. Thus, these findings indicate less working memory capacity available reduced the effect of having selected a correct or an incorrect object previously; nevertheless, it clearly was not fully eliminated. Similarly, the effect of target-exposure remained significant in both conditions as well, though its estimate was reduced in the high-load condition (low-load: B = 0.82, SE = 0.03, Z = 25.14, *p* < 0.001, odds ratio = 2.27; high-load: B = 0.66, SE = 0.03, Z = 20.62, *p* < 0.001, odds ratio = 1.93), indicating lower learning per trial in that condition.

Intriguingly, in the main analysis, even after accounting for the interactions of load with these two processes, there was still a significant main effect of condition (B = -0.44, SE = 0.22, Z = -2.02, *p* = 0.044, odds ratio = 0.64). This suggests that the effects of working memory load are not entirely mediated by the effects of gradual learning and last-encounter-accuracy.

## General discussion

This experiment used a dual-task manipulation to ask how explicit and implicit learning processes interact during cross-situational word learning, and whether any specific aspect of cross-situational word learning requires explicit cognitive resources. In particular, we examined two specific influences on the learning curve identified by prior work (Medina et al., 2011; Roembke et al., 2018; Roembke & McMurray, 2016; Trueswell et al., 2013): the last-encounter-accuracy effect, which has been attributed to hypothesis testing (propose-but-verify, posited to be an explicit strategy) and the target-exposure effect, a marker of (implicit) gradual learning. We found that increased memory load showed a relatively small overall effect on performance (Fig. 2B, Supplement S1 (OSM)). However, both trial-by-trial indices showed strong evidence for moderation by load: cognitive load lessened both the last-encounter-accuracy and the target-exposure effects. Finally, the effect of load was not limited to these effects – there was a small but significant main effect that even accounting for the interactions of load with last-encounter-accuracy and target-exposure.

We first consider the limitations of the experiment before turning to its implications for mechanisms of cross-situational word learning and learning more broadly.

### Limitations

First, participants in the high-load condition struggled more with completing the cover task than participants in the low-load condition. This was part of the design; nevertheless, it is possible that this manipulation of working memory also had unintended consequences on how participants performed on the word-learning trials. That is, they could have learned the mappings equally well but struggled to translate that knowledge to an accurate response on a given trial due to the memory load. As a result, any differences in accuracy cannot be interpreted as differences in word *learning*, but only as differences in how well word knowledge was executed.

Similarly, the load manipulation might have affected fatigue levels: That is, participants in the high-load condition might have become more tired as the experiment progressed, making it more difficult to form or select the correct word-object-mappings. Importantly, such cognitive fatigue effects might in fact be a sign that additional domain-general resources (instead of more automatic ones) are allocated to a task, thus requiring more sustained mental effort (e.g., mental fatigue as a result of hearing loss; Hornsby, 2013; McCoy et al., 2005). In such a scenario, fatigue might not be considered noise, but rather a potentially theoretically interesting consequence of our dual-task manipulation as a sign of increased load. But again, it is not clear whether the increased load (indicated by fatigue) leads to poorer learning or poorer performance.

However, given that we did not find strong evidence for a large overall performance difference in the word-learning analyses due to condition (e.g., Fig. 2, Supplement S1 (OSM)), such effects of the cover tasks were likely limited. Moreover, these concerns primarily affect overall performance, which was not of primary interest. Instead, our trial-by-trial analysis included a main effect of condition to account for general effects like these, while still isolating the effect of load on the two more specific indices. Nonetheless, this analysis still identified interactions of several factors with condition. Future experiments that target overall learning performance should include a subset of word learning trials without the memory task to investigate word-learning differences in the absence/presence of working memory resources more clearly.

Second, these results cannot speak to what other factors – over and above any explicit processes in working memory – contribute to cross-situational word learning or to the size of the last-encounter-accuracy effect or the effect of target-exposure. Our well-established dual-task/memory paradigm was able to examine the effect of limiting access to working memory resources on specific components of cross-situational learning. Nonetheless, the main effect of condition suggests there may be other aspects of word learning that are sensitive to load.

Third, working memory has been conceptualized differently by researchers, and it is possible that our results would have varied had we used a task based on a different definition of working memory (c.f., Cowan, 2017). Thus, to what extent other types of (explicit) processes contribute to cross-situational word learning should be the subject of future experiments.

Finally, we did not measure participants’ working memory capacity. As a result, we do not know if some people were more (or less) affected by our manipulation than others due to individual differences in working memory capacity. For example, a participant with a larger than average working memory might have been able to rely more on explicit encoding and maintenance of mappings, even if they were assigned to the high-load condition (see DeCaro, Thomas, & Beilock, 2008). Given that the participant pool was restricted to undergraduate students, it is likely that the range of working memory was relatively limited as well. However, had we measured working memory capacity, we may have been able to reduce the impact of this variable (noise) on our effects by either controlling for it statistically, or optimizing the load manipulation to the subjects’ own capacity (c.f., Otto et al., 2013).

### Interactions of explicit and implicit processes during cross-situational word learning

Participants were able to learn the set of word-object-mappings in both load conditions. However, performance was poorer when participants’ access to working memory resources was limited (e.g., the main effect of condition). In addition, the effect of the manipulation appeared stronger at the end of the experiment (the condition × target-exposure interaction; see Supplement S1 (OSM)). This offers evidence that the negative effect of limiting working memory availability was cumulative over time, though – as pointed out in the *Limitations* – it is unclear whether this performance difference was the result of differences in underlying knowledge of word-objects-mappings, participants’ ability to use the learned knowledge, or both. It is also consistent with the notion that early on, word learning is generally more driven by chance-level guessing (i.e., participants randomly clicking on one referent but not another) coupled with association building, whereas later it can take advantage of explicit processes (such as hypothesis testing or processes like mutual exclusivity; Roembke et al., 2018).

Despite this, it should also be pointed out that the effect of condition on overall performance was numerically small, suggesting that limiting access of working memory impacted cross-situational word learning less than one we originally anticipated. Rather, word learning in this paradigm is primarily implicit, with smaller potential gains on top of this due to explicit processing.

Our primary analysis found statistically robust interactions of load with two specific influences on trial-by-trial performance. We start by discussing the pattern of these effects independent of load condition, before turning to their moderation by load.

At face value, these two indices – last-encounter-accuracy and target-exposure – appear to have analogs in explicit and implicit processing. Last-encounter-accuracy has been agued to be a marker of an explicit inferencing process (proposing and verifying) (Dautriche & Chemla, 2014; Roembke & McMurray, 2016; Trueswell et al., 2013), whereas target-exposure has been argued to reflect gradual, statistical or associative learning (Dautriche & Chemla, 2014; Roembke & McMurray, 2016). Independent of load condition, these effects patterned similarly in this study to prior studies.

As in previous experiments, we found a significant interaction between last-encounter-accuracy and target-exposure: specifically, the effect of last-encounter-accuracy increased over the course of the experiment (Roembke & McMurray, 2016). This is perhaps consistent with a reduction in pure guessing (which is almost a necessity at the beginning of the experiment), in favor of decisions that are guided by other sources of knowledge. One possibility is episodic memory, which could nudge these guesses toward answers guided by the memory of previous trials. At this point, the role of episodic memory is not clear in this context: It could be that participants’ memories of past selections influence what object they choose on a current trial; however, it could also be that statistical information is weighed more strongly if it is “collected” more recently, thus resulting in a stronger effect of last-encounter-accuracy if a word was heard only a trial or two ago. These two mechanisms may be hard to distinguish, as they result in very similar if not identical predictions. The similarity of this pattern to prior work suggests that word learning in this experiment was not qualitatively different from learning in single-task designs (Roembke et al., 2018; Roembke & McMurray, 2016).

When we examined how the effect of memory load interacted with these trial-by-trial indices, there were several important findings. First, there was a main effect of condition, again reflecting overall greater difficulty under high load. Critically, this effect of load was found even after controlling for its possible mediation by factors like target-exposure and the last-encounter accuracy effect. This suggests that there may be other explicit processes that are not tapped by these markers, an important avenue for future research.

Second, participants in the high-load condition showed a reduced effect of last-encounter-accuracy (a significant last-encounter-accuracy × condition interaction). This was predicted on the basis that explicit processes may rely on working memory resources to “remember” what was last clicked on. However, this effect was not fully eliminated: Participants both in the low-load as well as in the high-load condition showed an effect of last-encounter-accuracy, even though it was reduced in the high-load condition. We see several possible explanations for this. One possibility is that the last-encounter-accuracy effect is driven only partially by explicit processing. For example, it may simply derive from the fact that associations between words and objects get more of a boost when the object is selected. This small boost when the object was correctly chosen on the prior trial then can then be amplified by competition among referents in the current trial (McMurray et al., 2012). This is consistent with the fact that pigeons – a species with little capacity for explicit processing (c.f., O’Donoghue et al., 2020) – also show an effect of last-encounter-accuracy (Wasserman et al., 2015). If this is true, this would challenge the utility of this as a unique marker of inferential processing.

Alternatively, the hypothesis testing described by propose-but-verify may be embedded in language areas and may not require domain-general resources. A third possibility is that the last-encounter-accuracy effect is fully explicit, and we simply did not load working memory sufficiently. This seems unlikely as five digits is a fairly large load. What is important is that our working memory manipulation reduced this effect – even controlling for the main effect of condition – suggesting that at least some portion of it requires domain-general resources.

Surprisingly, we also found that the target-exposure effect was also moderated by working memory load, and in the same direction as the last-encounter-accuracy effect: learners in the high-load condition showed a smaller gradual learning effect than those in the low-load condition. This was not expected. The exposure effect was predicted to reflect statistical or associative learning that does not require working memory resources, or that maybe would even benefit by blocking a competing explicit learning system. Instead, we found the opposite. One possibility is that statistical or gradual learning – or the input to it – is resource requiring. This is consistent with Toro, Sinnett, and Soto-Faraco (2005) and Turk-Browne, Jungé, and Scholl (2005), who both found reduced statistical learning under divided attention (though other studies report no such effect: Musz et al., 2014; Saffran et al., 1997). If these studies are right, then statistical learning may be gated by attention.

Importantly, the fact that both effects are moderated by memory load in the same direction suggests that the hypothesis testing represented by the last-encounter-accuracy effect and the gradual learning represented by the exposure effect do not operate in competition. If this were the case, one would have predicted the effect of target-exposure to be either unaffected by participants’ condition or even increased in the high-load condition when access to explicit processes was reduced. Instead, we found that target-exposure also interacted with condition, but that this was the result of a *reduction* of its effect in the high-load condition. That is, less statistical learning occurs when people have limited access to explicit processes.

Even though both effects were moderated by load, it is unlikely that they represent purely explicit processes. This is for three reasons. First, with 12 objects and referents, even if we assume only memory for a single hypothesis for each word, this would far exceed any reasonable estimate of capacity. Second, likely because of this, the overall influence of memory load was small despite a rather large memory load. This implies that both processes may be only partly explicit (resource requiring) and may be largely implicit. Finally, Warren et al. (2020) showed that patients with hippocampal amnesia can learn words cross-situationally, though with reduced performance relative to a control group. The hippocampus is often thought to be responsible for learning verbalizable rules (consistent with explicit processing) and “memory in the moment” (e.g., during a visual search task; Voss et al., 2011). Thus, together these results suggest unambiguous evidence for an implicit component.

Nonetheless, we should not dismiss explicit processing either. Even after accounting for the effect of memory load on the last-encounter-accuracy and target-exposure effects, there was still a main effect of condition. This implies that memory load can influence performance in a cross-situational word-learning task via other mechanisms than the two that we have identified and quantified here. Moreover, Berens, Horst, and Bird (2018) reported that hippocampus activity was more predictive of cross-situational word learning than activity in other brain areas. These findings suggest a clear explicit component as well. One way to consider the neuroscience is that that implicit processes might be more important for cross-situational learning when explicit processes are not an option (as in a dual-task paradigm or after hippocampal amnesia). However, our data suggest that such a view may underestimate the breadth of interactions that occur between explicit and implicit processes during cross-situational learning.

Taken together these data support a hybrid model in which both implicit and explicit processes play a role. Moreover, our data are consistent with a unique hybrid in which attention may play a critical role. In the McMurray et al. (2012) approach to word learning, true learning takes place slowly as associations are built between words and objects. However, in the moment, real-time competition processes direct attention to more likely referents (and away from incorrect mappings). Learning occurs on the “output” of these processes such that attended objects get more associative boost. Thus, anything that reduces attention may reduce the efficiency of this competition and impact learning. However, even if these competition processes are inefficient or fail (e.g., in patients with amnesia or in the high-load condition), associative learning can still occur. This then may explain how working memory load can influence both aspects of learning in the same direction.

Critically, this attentional manipulation can in principle affect both aspects of learning. If competition does not fully resolve, then more spurious associations (e.g., between the target and other objects) will be formed since these referents could not be fully suppressed in the moment. This would result in a smaller effect of target-exposure. This could lead to a smaller last-encounter-accuracy effect in one of two ways. First, recall that the last-encounter-accuracy effect interacts with target-exposure – it is bigger later in learning. This implies that last-encounter-accuracy is a product of learning – if learning is slowed, then it should be reduced. Second, if competition resolves less accurately, then the word-object-associations laid down on the last encounter will be less robust, and these in turn will be less amplified in the moment. Finally, given the pervasive nature of attention, this may also have other effects on performance that are not captured by these indices (accounting for the main effect).

Note that here the only explicit process necessary in this account is attention. However, the hybrid approach is consistent with other explicit processes as well. For example, working memory of prior trials, as well as inference and hypothesis testing could also guide attention to the correct object in the moment, and this can ultimately benefit learning. Critically, such an account acknowledges both implicit and explicit processes, but with only a single underlying system for storing word-object-mappings (a single learning system).

## Conclusions

Our study shows that working memory load affects two specific aspects of cross-situational word learning: both the effects of last-exposure-accuracy and target-exposure were reduced when participants learned words cross-situationally. Prior work has linked these to more explicit and implicit processes. However, our findings suggest that that these two processes do not compete (as predicted by classic dual-systems accounts of learning); rather they support each other. As a result, they are unlikely to derive from independent learning systems. Instead, cross-situational learning may be a product of more basic associative learning mechanisms buttressed by in-the-moment attention.

## Supplementary Information

ESM 1 (DOCX 56 kb)

## Footnotes

1 Note that we did not also employ the confidence measures used in this study because we were worried that the slower pace they require would disrupt the trial-by-trial indices essential to the logic of the present study.

2 Each trials’ target object was equally likely to appear in one of the four locations. To quantify motivation, we scored a trial as “1” if participants had clicked the object in the same location as on the preceding trial. We then computed the proportion of trials in which participants had clicked in the same location as before. On average, participants clicked in the same location as the preceding trial on 0.25 of trials. Participants who were excluded (proportion was 2SD above average) clicked in the same location as before on 0.53 of trials or above.

3 The empirical logit transformation was applied to stretch the space near 0 and 1 to overcome differences in the learning curve (there is more numerical room to improve when one is in a medium accuracy range than when close to ceiling levels).
