# Supplementary material for: Multiple components of statistical word learning are resource dependent: Evidence from a dual-task learning paradigm
Source: Mem Cognit. 2021 Mar 17;49(5):984–97. doi: 10.3758/s13421-021-01141-w (PMC8238696; doi:10.3758/s13421-021-01141-w)
Supplement: Supplementary file 1 — (DOCX 56 kb) [file 13421_2021_1141_MOESM1_ESM.docx]

**Multiple components of statistical word learning are resource dependent:**

**Evidence from a dual task learning paradigm.**

Tanja C Roembke

Institute of Psychology

RWTH Aachen University

And

Bob McMurray

Dept. of Psychological and Brain Sciences

Dept. of Communication Sciences and Disorders

Dept. of Linguistics

University of Iowa

**Supporting Materials S1: Overall analysis of accuracy**

Figure S1 shows overall accuracy in the cross-situational learning trials as a function of load condition and block (of 120 trials). It suggests that learners learned better in the low-load condition, and that this difference may have increased with time. While the primary analysis of this data used the trial-by-trial approach, here we present an analysis of overall accuracy, similar to other dual-task learning studies.

*Figure S1.* Word learning accuracy across blocks by condition*.* Each block represents 120 cross-situational learning trials. Error bars reflect SEM. Reprinted from Figure 2B in the main text.

To investigate this statistically, binomial mixed effect models were implemented in R (version Ri386 3.6.3; R Core Team, 2014) with the lme4 (Bates & Maechler, 2009), and nlme (Pinheiro et al., 2020) packages. The dependent variable was accuracy. Fixed effects consisted of condition (high-load/low-load [+/-0.5]), block (1-3 [120 trials each]; centered) and their interaction. To provide a measure of effect size for each reported effect in the selected models, we calculated odds ratios by exponentiating the raw coefficients.

To identify the random effects, a series of nested models were compared to find the model with most complex random effect structure required to fit the data (Matuschek et al., 2017), holding the fixed effects constant. We used a forward selection approach, evaluating more complex model against a less complex model using the chi-square test of model comparison.

The models that were evaluated are presented in Table S1. We first compared nested Models 1-3 which used random intercepts of three possible effects: subject, word (the word played on that trial), and target object (the visual stimulus corresponding to the word). Since words were randomly assigned to objects for each subject, the object and the word could serve as unique effects in the model.

Model 3 captured the data better than Models 1-2 (*p* < 0.001). Subsequently, we ran Models 4-6 (using Model 3 as a starting point) where we evaluated increasingly complex random slope structures (note that it was not possible to add condition as a random slope as it differed between subjects). Only Model 4 converged, and it better accounted for the data than Model 3 (*p* < 0.001). This model was thus used for inference.

This model included a random slope of block on subject, and random intercepts of target word and target object. In this model, the main effect of block was significant (B = 1.15, SE = 0.12, Z = 9.27, *p* < 0.001, odds ratio = 3.16), indicating that participants became more accurate as the experiment proceeded. In addition, there was a marginally significant main effect of condition (B = -0.60, SE = 0.35, Z = -1.68, *p* = 0.093, odds ratio = 0.55), and a marginally significant interaction between block and condition (B = -0.46, SE = 0.25, Z = -1.85, *p* = 0.065, odds ratio = 0.63).

In the interest of full disclosure, we note that for the initial submission of this manuscript we first ran these analyses in an older version of R (version Ri386 3.6.1), and Model 4 did not converge. Thus, this earlier version reported results from Model 3 (the most complex model that still converged). Using Model 3, the main effect of block was significant (B = 0.78, SE = 0.02, Z = 42.45, *p* < 0.001, odds ratio = 2.18), indicating that participants became more accurate as the experiment proceeded. In addition, there was a significant main effect of condition (B = -0.51, SE = 0.26, Z = -1.96, *p* = 0.0498, odds ratio = 0.60), and a significant interaction between block and condition (B = -0.36, SE = 0.04, Z = -9.86, *p* < 0.001, odds ratio = 0.70).

*Table S1:* Models evaluated during word learning analysis. All evaluated models included fixed effects of condition (high-load/low-load), block (1-3; centered) and their interaction.

| **Model** | **Random effect structure** | **Comparison with previous** | **Notes** |
| --- | --- | --- | --- |
| 1 | (1\|Subject) | ̶ |  |
| 2 | (1\|Subject) + (1\|Word) | *χ^2^*(1) = 4.90,  *p* = 0.027 |  |
| 3 | (1\|Subject) + (1\|Word) + (1\|Target Object) | *χ^2^*(1) = 43.31  *p* < 0.001 | Selected after first evaluation |
| 4 | (Block \|Subject) + (1\|Word) +  (1\|Target Object) | *χ^2^*(2) = 1671  *p* < 0.001 | Selected after second evaluation |
| 5 | (Block \|Subject) + (Block \|Word) + (1\|Target Object) | ̶ | Did not converge |
| 6 | (Block \|Subject) + (Block \|Word) +  (Block \|Target Object) | ̶ | Did not converge |

These results suggest that the higher working memory load affected participants’ word learning performance only to a very small extent, though there was a trend for word learning performance to be lower in the high-load than in the low-load condition: Accuracy tended to be lower when participants had to hold more digits in their working memory, and this appears to be driven by performance differences late in the experiment (see Figure S1).

Note that we report much more robust effect of condition in the main analyses – with both a significant main effect of condition and interactions with last-encounter-accuracy and target-exposure. These effects were likely more robust than these overall analyses because these trial-by-trial analyses accounted for more sources of nuisance variance (item effects, last-encounter effects), and treated exposure as continuous (not blocked).

**Supporting Materials S2: Model selection for Trial-by-Trial Analysis.**

This section describes our model selection strategy for the primary analysis of word learning: the trial-by-trial analysis. As described in the main manuscript, the fixed effects were last-encounter-correct (1/0), target exposure (log scaled), condition and all their possible interactions. To find the random effect structure for the baseline model, we first ran the models without condition to facilitate convergence. This was possible, as condition is a between-subject variable. The models that were evaluated are given in Table S2.

We first evaluated Models 1-3 (all nested) to determine which random intercepts were needed. This indicated that Model 2 was not better at capturing the data than Model 1, but Model 3 was. This indicates that target object as a random object was helpful in capturing the data pattern, but not the random stimulus for word. Therefore, we constructed Model 4 and compared it with Model 1; it was found that Model 4 significantly better captured the data (*χ^2^*(1) = 16.00, *p* < 0.001). Thus, we used it as the basis of our additional model explorations. However, none of the more complex models we constructed converged (see Table S2). (Note that given that Models 5 and 6 were not nested, they were compared separately to Model 4.)

We then added condition as well as all the resulting interactions as fixed factors to create a new model and compared it to Model 4. This was found to significantly improve data fit (*χ^2^*(4) = 105.15, *p* < 0.001). The resulting model is what used in the main manuscript (described there in equation 1).

*Table S2:* Models evaluated during the trial-by-trial analyses. All evaluated models included fixed effects of last-encounter-correct, target exposure and its interaction.

| Model | Random effect structure | Comparison with previous model | Notes |
| --- | --- | --- | --- |
| 1 | (1\|Subject) | ̶ |  |
| 2 | (1\|Subject) + (1\|Word) | *χ^2^*(1) = 0.91,  *p* = 0.339 |  |
| 3 | (1\|Subject) + (1\|Word) + (1\|Target Object) | *χ^2^*(1) = 15.94,  *p* < 0.001 |  |
| 4 | (1\|Subject) + (1\|Target Object) | *χ^2^*(1) = 16.00,  *p* < 0.001 | Compared to Model 1; selected after second evaluation |
| 5 | (last-encounter-correct * target exposure \|Subject) + (1\|Target Object) | ̶ | Did not converge |
| 6 | (last-encounter-correct + target exposure \|Subject) + (1\|Target Object) | ̶ | Did not converge |

**References**

Matuschek, H., Kliegl, R., Vasishth, S., Baayen, R. H., & Bates, D. M. (2017). Balancing type I error and power in linear mixed models. *Journal of Memory and Language*, *94*(2013), 305–315. https://doi.org/10.1016/j.jml.2017.01.001

Pinheiro J, Bates D, DebRoy S, Sarkar D, R Core Team (2020). nlme: Linear and Nonlinear Mixed Effects Models. R package version 3.1-150, <https://CRAN.R-project.org/package=nlme>.

R Core Team (2014). R: A language and environment for statistical computing. R Foundation for Statistical Computing, Vienna, Austria. URL <http://www.R-project.org/>.
